# Periplocin improves the sensitivity of oxaliplatin-resistant hepatocellular carcinoma cells by inhibiting M2 macrophage polarization

**DOI:** 10.17305/bb.2024.10928

**Published:** 2024-08-27

**Authors:** Jiefeng Weng, Hui Liu, Zhaofeng Wu, Yu Huang, Shuai Zhang, Yujie Xu

**Affiliations:** 1 Department of Hepatobiliary Pancreatic Surgery, Guangzhou First People’s Hospital, Guangzhou City, China; 2 Physical Examination Center, Guangzhou First People’s Hospital, Guangzhou City, China

**Keywords:** Periplocin, oxaliplatin, hepatocellular carcinoma, M2 macrophage polarization, chemotherapy resistance

## Abstract

The aim of this research was to investigate the impact of periplocin (PPLN) on oxaliplatin (OXA) resistance in hepatocellular carcinoma (HCC) cells and offer insights for improving clinical treatment of HCC. The IC50 value of HCC cell lines against OXA was detected by the CCK-8 assay, and an OXA-resistant HepG2 cell line (HepG2/OXA) was constructed. THP-1 cells were induced into M1 or M2 macrophages, and M2 macrophage-conditioned medium (M2-CM) was prepared. M1 and M2 macrophage polarization were detected using RT-qPCR and flow cytometry. CCK-8, EdU staining, clone formation assay, flow cytometry, and western blotting were used to assess the proliferation and apoptosis of HepG2/OXA cells treated with PPLN and M2-CM. Additionally, a nude mouse subcutaneous graft tumor model was constructed. PPLN enhanced the sensitivity of HepG2/OXA cells to OXA, reduced their clone-forming ability, and promoted their apoptosis. Notably, PPLN hindered M0 macrophage polarization to M2 macrophages, while M1 polarization remained unaffected. The proliferation-inhibiting and apoptosis-promoting effects of OXA+PPLN on HepG2/OXA cells were significantly attenuated by the addition of M2-CM, suggesting that PPLN improves the OXA sensitivity of HepG2/OXA cells by hindering M2 macrophage polarization. Furthermore, PPLN inhibited M2 macrophage polarization and improved the OXA sensitivity of HepG2/OXA cells *in vivo*. In conclusion, PPLN inhibited the proliferation of HepG2/OXA cells, promoted their apoptosis, and inhibited M2 macrophage polarization both *in vivo* and *in vitro*, which in turn enhanced the OXA sensitivity of HepG2/OXA cells.

## Introduction

Hepatocellular carcinoma (HCC) ranks as the fifth most prevalent malignant tumor globally and makes up 90% of all primary liver cancers [1, 2]. According to statistics, the five-year survival rate for HCC patients in Asian countries is a mere 18%, plummeting to 2% for those with advanced HCC [3, 4]. At early diagnosis, treatment options for HCC include surgical resection, liver transplantation, and local ablation [5, 6]. However, the majority of individuals with HCC come to the clinic when the tumor is already at an advanced stage and cannot be surgically removed. Chemotherapy is one of the mainstays of treatment for this group of patients, with platinum-based drugs having been commonly used in HCC treatment [7, 8].

Cisplatin, a first-generation platinum-based drug, has become a major chemotherapeutic agent for the treatment of various solid tumors [9, 10]. The second-generation platinum-based anticancer drug carboplatin is structurally very similar to cisplatin, and its mechanism of action is also similar to cisplatin. It is used as well for the treatment of various tumors [11]. Oxaliplatin (OXA), a third-generation platinum-based anticancer drug, works in a similar way to other platinum-based drugs. The platinum atoms in OXA interact with DNA to block DNA replication and transcription [12, 13]. OXA has better therapeutic efficacy and higher tolerability than other platinum-based drugs, and OXA-based chemotherapy has been found to be beneficial for patients with advanced HCC [14, 15]. Although the therapeutic efficacy of platinum-based drugs for HCC has been recognized, HCC cells are highly susceptible to developing resistance to them, and the actual benefit to patients is very limited [16–18]. Therefore, finding ways to reverse the resistance of HCC to platinum-based drugs has become a challenging and important part of HCC treatment.

Periplocae displays a range of pharmacological properties, including cardiotonic, anti-tumor, and anti-inflammatory effects, with its cardiotonic effect mainly determined by cardiac glycosides [19]. Periplocin (PPLN), a cardiac glycoside, is the main active ingredient of Periplocae, which inhibits the malignant advancement of various cancer types, such as colorectal, gastric and pancreatic cancers [20–22]. Bae et al. [23] reported that PPLN hindered the proliferation of pancreatic cancer cells, leading to cell cycle arrest and apoptosis, and effectively increased the gemcitabine sensitivity of pancreatic cancer-resistant cells. Notably, Lin et al. [24] found that PPLN inhibited the AKT/NF-κB pathway in HCC cells, which in turn inhibited cell proliferation, induced cycle arrest, and promoted apoptosis. However, the role of PPLN in HCC resistance to OXA is currently unclear.

Macrophages are major cells that exert immune functions. Several studies have shown the significant impact of tumor-associated macrophage M2 polarization on drug resistance in tumor cells [25, 26]. Therefore, we initiated this research by establishing a HepG2/OXA-resistant cell line and investigating the effects of PPLN and M2 macrophage polarization on HepG2/OXA cells. Next, we explored whether PPLN affects M0 macrophage polarization toward M1 or M2, and further investigated whether PPLN enhances the OXA sensitivity of HepG2/OXA cells by modulating M2 macrophage polarization. Finally, a nude mouse subcutaneous graft tumor model was constructed to investigate the effects of PPLN and OXA on tumors in nude mice *in vivo*. The aim of this research was to examine the impact of PPLN on OXA resistance in HCC cells and offer insights for improving the clinical treatment of HCC.

## Materials and methods

### Cell culture

Human HCC cell lines (Huh-7 and HepG2) and human leukemia mononuclear cells (THP-1) were purchased from Pricella Biotechnology Co., Ltd. (Wuhan, Hubei, China). Human HCC cells (MHCC-97H) were obtained from the Shanghai Cell Bank, Chinese Academy of Sciences. THP-1 cells were grown in RPMI-1640 medium (Gibco, Grand Island, NY, USA) containing 0.05 mM β-mercaptoethanol (Gibco), 10% fetal bovine serum (Gibco), and 1% penicillin/streptomycin double antibody (Gibco). Huh-7, HepG2, and MHCC-97H cells were grown in DMEM medium (Gibco) containing 10% fetal bovine serum and 1% penicillin/streptomycin double antibody. All cell cultures were maintained at 37 ^∘^C with 5% CO_2_.

### Establishment of the HepG2/OXA drug-resistant cells

HepG2 cells were seeded in six-well plates and exposed to 5 µM OXA for 24 h after adherence, and then cultured in DMEM complete medium (without OXA). Once the cells recovered to a good state, they were treated with increasing concentrations of OXA treatment according to a gradient concentration. The concentrations of OXA used were as follows: 10, 15, 20, 30, 40, 50, 70, 85, and 100 µM. After culturing the cells continuously for approximately six months in this method, a high-concentration drug-resistant cell line, HepG2/OXA, was obtained. This cell line was able to maintain a good growth state in medium containing 30.0 µM OXA. For subsequent functional experiments, drug-resistant cells were first cultured under OXA-free conditions for more than a week to exclude the residual effects of OXA.

### Preparation of M2 macrophage-conditioned medium

THP-1 cells were exposed to 100 ng/mL PMA (HY-18739, MedChemExpress, Monmouth Junction, NJ, USA) for 24 h to induce differentiation into M0 macrophages. M0 macrophages were then exposed to lipopolysaccharides (LPS, 20 ng/mL, HY-D1056, MedChemExpress) and interferon-γ (IFN-γ, 20 ng/mL, HY-P7025, MedChemExpress) for 48 h to induce M1 macrophages. M0 macrophages were induced into M2 macrophages by stimulation with interleukin-4 (I4269, IL-4, 20 ng/mL, Sigma-Aldrich, St. Louis, MO, USA) and interleukin-13 (I1771, IL-13, 20 ng/mL, Sigma-Aldrich) for 48 h. The supernatant was collected by centrifugation and designed as M2 macrophage-conditioned medium (M2-CM).

### CCK-8 assay

HCC cells, M1 macrophages and M2 macrophages were seeded in 96-well cell culture plates (1.5 × 10^4^/well). After attachment to the wall, the original medium was replaced with 200 µL of medium containing OXA (2.5, 5, 10, 20, 40, and 80 µM, HY-17371, MedChemExpress), PPLN (20, 40, 80, 150, and 300 nM, HY-N1381, MedChemExpress) or M2-CM (1:1 ratio with DMEM medium). Following a 48-h incubation period, 20 µL of CCK-8 reagent (C0038, Beyotime, Shanghai, China) was added to each well. After incubating for 2 h at 37 ^∘^C in a light-protected incubator, the OD_450_ value was measured using a microplate reader (Thermo Fisher Scientific, Waltham, MA, USA). Based on the inhibition rate of cell viability by OXA and PPLN, CalcuSyn software 2.0 was used to calculate the combination index (CI) of the combined drugs as a way to determine whether the drugs were synergistic or not.

### EdU staining

The EdU Cell Proliferation Detection Kit (C0071S, Beyotime) was used to assess the proliferation of HCC cells. Cells from different treatments were cultured for 48 h. After being rinsed twice with PBS, the cells were exposed to a 10 µM EdU culture solution for 1 h in the dark. The cells were then washed twice with PBS and fixed with 4% paraformaldehyde (Solarbio, Beijing, China) for 15 min. To permeabilize the cells, PBS containing 0.3% Triton X-100 (Sigma-Aldrich, St. Louis, MO, USA) was added for 10 min. Next, Click reaction solution (Invitrogen, Carlsbad, CA, USA) was applied and incubated for 30 min in a light-free environment. DAPI staining solution (Invitrogen) was then applied for approximately 10 min. After anti-quenching sealing, the cells were observed and photographed under a fluorescence microscope.

### Clone formation assay

Cells from each group in the logarithmic growth phase were collected, washed with PBS, digested with 0.25% trypsin (Gibco), and dispersed into single cells, which were then counted. Five hundred cells were placed in each well of a six-well cell culture plate, and incubated for 14 days (37 ^∘^C, 5% CO_2_). The culture was terminated when clonal cell clusters were visible to the naked eye. The culture solution was aspirated, and the cells were rinsed twice with PBS. Next, 4% paraformaldehyde was added for fixation for 20 min. The fixative was discarded, and the cells were stained with crystal violet (Sigma-Aldrich) for 15 min and then photographed for counting.

### Flow cytometry

Cells from different treatments were cultured for 48 h, rinsed twice with PBS, and gently mixed by adding 500 µL of Binding Buffer. Annexin-V-FITC (5 µL, MedChemExpress) and propidium iodide (5 µL, Beyotime) were then added, gently mixed, protected from light, and incubated for 15 min. Flow-specific supersampling tubes were used to transfer the samples, and apoptosis was analyzed using flow cytometry (BD FACSCaliburTM, BD Biosciences, San Jose, CA, USA).

### RT-qPCR

Total RNA was isolated from different sets of THP-1 cells using Trizol reagent (Invitrogen). Reverse transcription was performed by adding AMV reverse transcriptase (Takara, Tokyo, Japan) to obtain cDNAs. PCR amplification was carried out using TB Green® Premix Ex Taq™ II (TAKARA). Using β-actin as an internal reference, the relative expression of target genes was determined using the 2^−ΔΔCt^ method.

The primer sequences used in this experiment are as follows: CD206: F: 5′-GCCTCGTTGTTTTGCGTCTT-3′; R: 5′-CCCCGTTTCCTCCTCACAAA-3′. Interleukin-10 (IL-10): F: 5′-CCCTGCAATCAGGAAGCAGA-3′; R: 5′-AGGGCATCAAAAAGACCGCA-3′. Arginase-1 (Arg1): F: 5′-ACCTGAAACCAAGTCCCAGC-3′; R: 5′-CGAGCAAGTCCGAAACAAGC-3′. Fizz1: F: 5′-CGTAGTCGCCATTCCCTCTC-3′; R: 5′-ACACAGTGTCACCTCCAAGG-3′. CD86: F: 5′-TCCAGGCACTGTGCTAAACAT-3′; R: 5′-ACTAGCTCAAAACCCTGGCA-3′. Inducible nitric oxide synthase (iNOS): F: 5′-CAGCATGAGCCCCTTCATCA-3′; R: 5′-TGAAGTCTGTGTCCGAAGGC-3′. Tumor necrosis factor-α (TNF-α): F: 5′-GGAAGAGGTGAGTGCCTGG-3′; R: 5′-GCCCTGAGGTGTCTGGTTTT-3′. Interleukin-6 (IL-6): F: 5′-GCTTCCCTCAGGATGCTTGT-3′; R: 5′-ATTAACTGGGGTGCCTGCTC-3′. β-actin: F: 5′-TCCTATGGGAGAACGGCAGA-3′; R: 5′-TCCTTTGTCCCCTGAGCTTG-3′.

### Macrophage polarization marker assay

THP-1 cells were seeded in six-well plates and rinsed once with sterile PBS after different treatments. Cells were collected by centrifugation and resuspended in sterile PBS to prepare a single-cell suspension (1.0 × 10^7^/mL). PE-labeled CD206 antibody (12-2061-82, Invitrogen) and FITC-labeled CD86 antibody (11-0862-82, Invitrogen) were added, mixed, and incubated for 30 min in the dark. The samples were transferred to a flow cytometer for analysis, and the experimental results were processed and visualized using FlowJo software (v10.8, BD biosciences).

### Western blot

RIPA lysate (P0013B, Beyotime) was used to lyse cells or tissues for protein extraction, and the BCA kit (P0012, Beyotime) was used to assess protein concentrations. The proteins were transferred to PVDF membranes (Invitrogen) after electrophoresis. Following rinsing, the membranes were incubated overnight at 4 ^∘^C with Bax primary antibody (MA5-14003, 1:100, Invitrogen) or Caspase 3 primary antibody (ab32351, 1:5000, Abcam, Cambridge, MA, USA). The next day, after being rinsed three times, the membranes were cultured with goat anti-rabbit IgG secondary antibody (31460, 1:10,000, Invitrogen). After exposure and development, the grayscale values of each protein band were assessed using ImageJ software, with β-actin (MA1-140, 1:5000, Invitrogen) serving as the internal reference.

### Tumor-bearing assay *in vivo*

Balb/c female nude mice were purchased from Vitalriver (Beijing, China) at 4–6 weeks of age. The mice were kept in a stable environment at a temperature of 22 ^∘^C and humidity ranging from 55% to 60%, following a 12-h light and dark cycle. Nude mice were injected subcutaneously with 0.2 mL of HepG2/OXA cell suspension (5.0 × 10^6^ cells/mouse) and randomly divided into Control, OXA, PPLN, and OXA+PPLN groups. Treatment began on the seventh day after inoculation: the OXA group received 10 mg/kg OXA intraperitoneally, the PPLN group received 15 mg/kg PPLN intraperitoneally, the OXA + PPLN group received 10 mg/kg OXA + 15 mg/kg PPLN intraperitoneally, and an equal amount of substrate solution received the Control group. The drugs were administered once daily for seven days. Subcutaneous tumor dimensions were measured on days 7, 14, 21, and 28 using a vernier caliper. On day 28, the mice were executed under anesthesia, and the tumors were excised and weighed. The study adhered to the guidelines approved by the Guangzhou Meyers Biotechnology Co., LTD. Laboratory Animal Center.

### TUNEL staining

Tumor tissues from nude mice were exposed to 4% paraformaldehyde, routinely dehydrated, sectioned after paraffin embedding (thickness of 4∼5 µm), and deparaffinized with xylene (Sigma-Aldrich). Gradient ethanol hydration (100%, 95%, 75%, and 50%) was performed for 5 min each. DNase-free proteinase K (20 µg/mL, ST532, Beyotime) was slowly added, followed by a 30-min incubation and three washes with PBS. TUNEL assay solution (C1086, Beyotime) was carefully applied and incubated for 1.5 h away from light. Subsequently, DAPI staining solution was added and incubated for 10 min. Samples were observed under a fluorescence microscope, and photographs were taken.

### Immunohistochemistry

Tumor tissue was fixed using 4% paraformaldehyde, routinely dehydrated, embedded in paraffin, and sections were prepared. Sections were dried, dewaxed, hydrated, microwaved for antigen retrieval, and treated with 3% H_2_O_2_ to inactivate endogenous peroxidase. Bovine serum albumin (Sigma-Aldrich) was used for blocking for 30 min. The blocking solution was removed, and CD206 primary antibody (PA5-82136, 1:1000, Invitrogen), Arg1 primary antibody (PA5-29645, 1:1000, Invitrogen), CD86 primary antibody (MA1-10293, 1:1000, Invitrogen), or iNOS primary antibody (PA1-036, 1:20, Invitrogen) was added dropwise and incubated at 4 ^∘^C overnight. On the following day, the secondary antibody was incubated at room temperature for 1 h. The color was developed using DAB solution (Solarbio), and the reaction was terminated in distilled water. Mayer hematoxylin (Sigma-Aldrich) was used for re-staining, and the film was sealed with a neutral dendrimer. The samples were observed under an inverted microscope, and photographs were taken.

**Figure 1.**
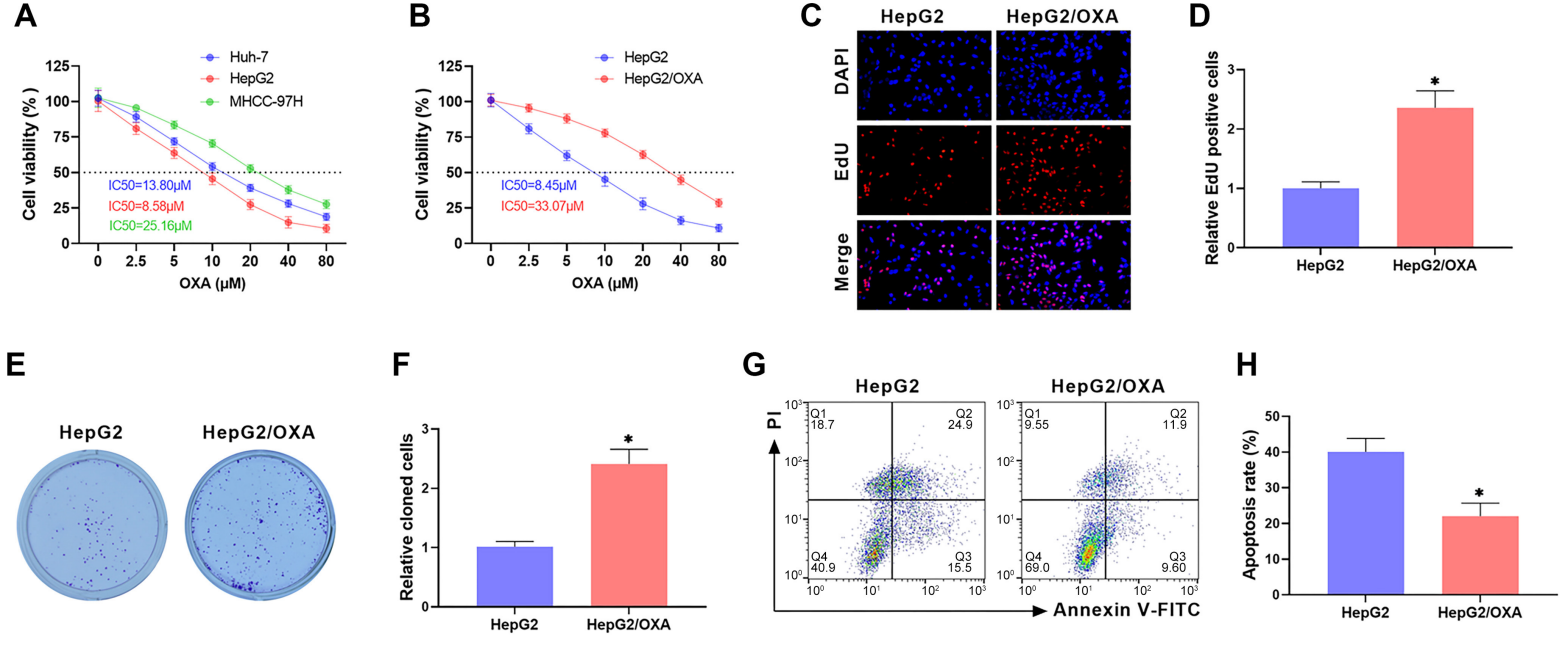
**Construction and validation of OXA-resistant HCC cell lines.** (A and B) The IC50 of Huh-7, HepG2, MHCC-97H cells, and HepG2/OXA cells against OXA was assessed using the CCK-8 assay; (C and D) EdU staining was utilized to determine the influence of OXA (10 µM) on HepG2 and HepG2/OXA cell proliferation; (E and F) Clone formation assay was used to evaluate the impact of OXA (10 µM) on clone formation in HepG2 and HepG2/OXA cells; (G and H) The apoptosis rate of HepG2 and HepG2/OXA cells after OXA (10 µM) treatment was determined using flow cytometry. HCC: Hepatocellular carcinoma; HepG2/OXA: OXA-resistant HepG2 cell line; OXA: Oxaliplatin.

### Immunofluorescence

Paraffin sections were deparaffinized with xylene, hydrated with gradient ethanol, and subjected to antigen retrieval. Tissue sections were permeabilized with a drop of 0.3% Triton X-100 (Sigma-Aldrich) for 10 min, and blocked by adding bovine serum albumin for 2 h. CD206 antibody (1:500) or CD86 antibody (1:200) was added and incubated overnight at 4 ^∘^C. On the next day, FITC-labeled secondary anti-goat anti-rabbit IgG (1:10,000) was added and incubated for 1 h at 37 ^∘^C away from light. Finally, DAPI staining solution was added and incubated for 10 min at room temperature, also protected from light. Observations were made with an inverted fluorescence microscope.

### Ethical statement

The study adhered to guidelines approved by Guangzhou Meyers Biotechnology Co., LTD. Laboratory Animal Center.

### Statistical analysis

Every experiment was performed at least three times, and the results are presented as the mean value ± corresponding standard deviation. SPSS 26.0 software (IBM SPSS Statistics 26) was used for statistical processing and analysis. Student’s *t*-test was used to evaluate differences between the two groups, while ANOVA was used for comparisons among sub-multiple groups. Prism software (GraphPad 9.0) was used for plotting. *P < 0.05 signifying that there was a significant difference.

## Results

### Construction and validation of OXA-resistant HCC cell lines

Previous studies have shown that Huh-7, HepG2, and MHCC-97H cells are commonly used HCC cell lines for studying OXA resistance [27, 28]. The IC50 of different HCC cell lines against OXA was determined using the CCK-8 assay, and the findings revealed that the IC50 values of Huh-7, HepG2, and MHCC-97H cells were 13.80, 8.58, and 25.16 µM, respectively (Figure 1A). These results showed that HepG2 cells are the most sensitive to OXA, so we selected HepG2 cells to construct an OXA-resistant cell line (HepG2/OXA). The IC50 value of HepG2/OXA cells was 33.07 µM, approximately four times higher than the IC50 value of HepG2 cells (8.45 µM), suggesting successful construction of the HepG2/OXA-resistant cell line (Figure 1B). Since the IC50 value of Huh-7 cells was 8.45 µM, we treated HepG2 and HepG2/OXA cells with 10 µM OXA in subsequent experiments. EdU staining results showed a noticeable decrease in EdU positivity in HepG2 cells compared to HepG2/OXA cells, indicating that OXA’s inhibition of HepG2 cell proliferation was significantly greater than its effect on HepG2/OXA cells (Figure 1C and 1D). The clone formation experiments demonstrated a marked increase in the number of clones in HepG2/OXA cells compared to HepG2 cells after OXA treatment (Figure 1E and 1F). Additionally, flow cytometry indicated a notable increase in the apoptosis rate in HepG2/OXA cells compared to HepG2 cells following OXA treatment (Figure 1G and 1H). These results further confirmed the successful construction of HepG2/OXA-resistant cells.

**Figure 2.**
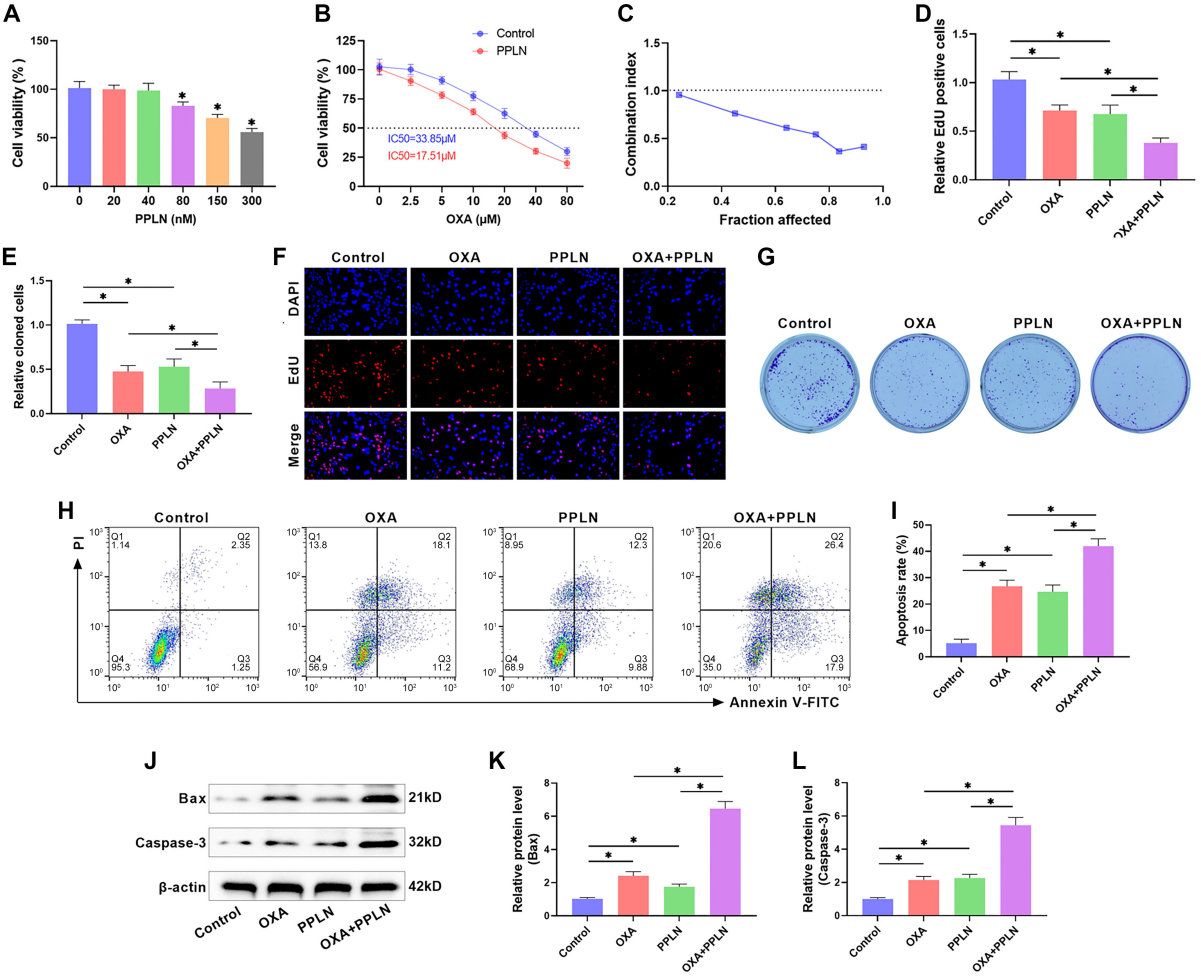
**PPLN enhances the sensitivity of HepG2/OXA cells to OXA.** (A) The impact of various doses of PPLN on the viability of HepG2/OXA cells was evaluated using the CCK-8 assay; (B) The IC50 of HepG2/OXA cells against OXA in the presence or absence of PPLN (80 nM) was assessed using the CCK-8 assay; (C) The combination index was calculated based on the inhibition rate of cell viability by PPLN and OXA to determine whether the combination therapy of OXA and PPLN is synergistic; (D and F) The proliferation of HepG2/OXA cells treated with OXA (10 µM), PPLN (80 nM), or OXA+PPLN was detected using EDU staining; (E and G) Colony formation assay was used to examine the influence of OXA (10 µM), PPLN (80 nM), or OXA+PPLN combination treatment on colony formation in HepG2/OXA cells; (H and I) The apoptosis rate of HepG2/OXA cells after OXA (10 µM), PPLN (80 nM), or OXA+PPLN co-treatment was quantified using flow cytometry; (J and K) Western blot analysis was used to examine the levels of Bax and Caspase 3 in HepG2/OXA cells following treatment with OXA (10 µM), PPLN (80 nM), or OXA+PPLN. PPLN: Periplocin; HCC: Hepatocellular carcinoma; HepG2/OXA: OXA-resistant HepG2 cell line; OXA: Oxaliplatin.

### PPLN enhances OXA sensitivity in HepG2/OXA cells

The activity of HepG2/OXA cells was assessed using the CCK-8 assay to evaluate the impact of PPLN. The results revealed a marked reduction in cell activity at PPLN concentrations of 80, 150, and 300 nM, with the effect being concentration-dependent (Figure 2A). For subsequent experiments, we chose 80 nM PPLN to treat the cells. Additionally, PPLN treatment enhanced the sensitivity of HepG2/OXA cells to OXA, as evidenced by a decrease in the IC50 value to 17.51 µM (Figure 2B). Notably, the CI was less than one after 48 h of OXA and PPLN co-treatment, suggesting a synergistic inhibitory effect of OXA and PPLN on HepG2/OXA cell proliferation (Figure 2C). EdU staining results showed that both OXA and PPLN treatments significantly reduced EdU positivity in HepG2/OXA cells, with the OXA+PPLN treatment being significantly more effective than either treatment alone (Figure 2D and 2F). The results of clone formation experiments showed that OXA or PPLN treatments significantly reduced the number of clones in HepG2/OXA cells, with OXA+PPLN treatment leading to a further reduction in the number of cloned cells (Figure 2E and 2G). Flow cytometry results indicated that OXA or PPLN treatments significantly reduced the apoptosis rate of HepG2/OXA cells, while OXA+PPLN treatment led to a further reduction in apoptosis rate (Figure 2H and 2I). Not only that, western blot analysis showed that the levels of apoptosis-related proteins Bax and Caspase-3 were markedly elevated in HepG2/OXA cells after OXA or PPLN treatments, and OXA+PPLN treatments led to a further elevation of their expression (Figure 2J and 2K). These findings indicate that PPLN enhances the effectiveness of OXA and increases the sensitivity of HepG2/OXA cells to OXA.

### M2 macrophages increase OXA resistance in HepG2/OXA cells

**Figure 3.**
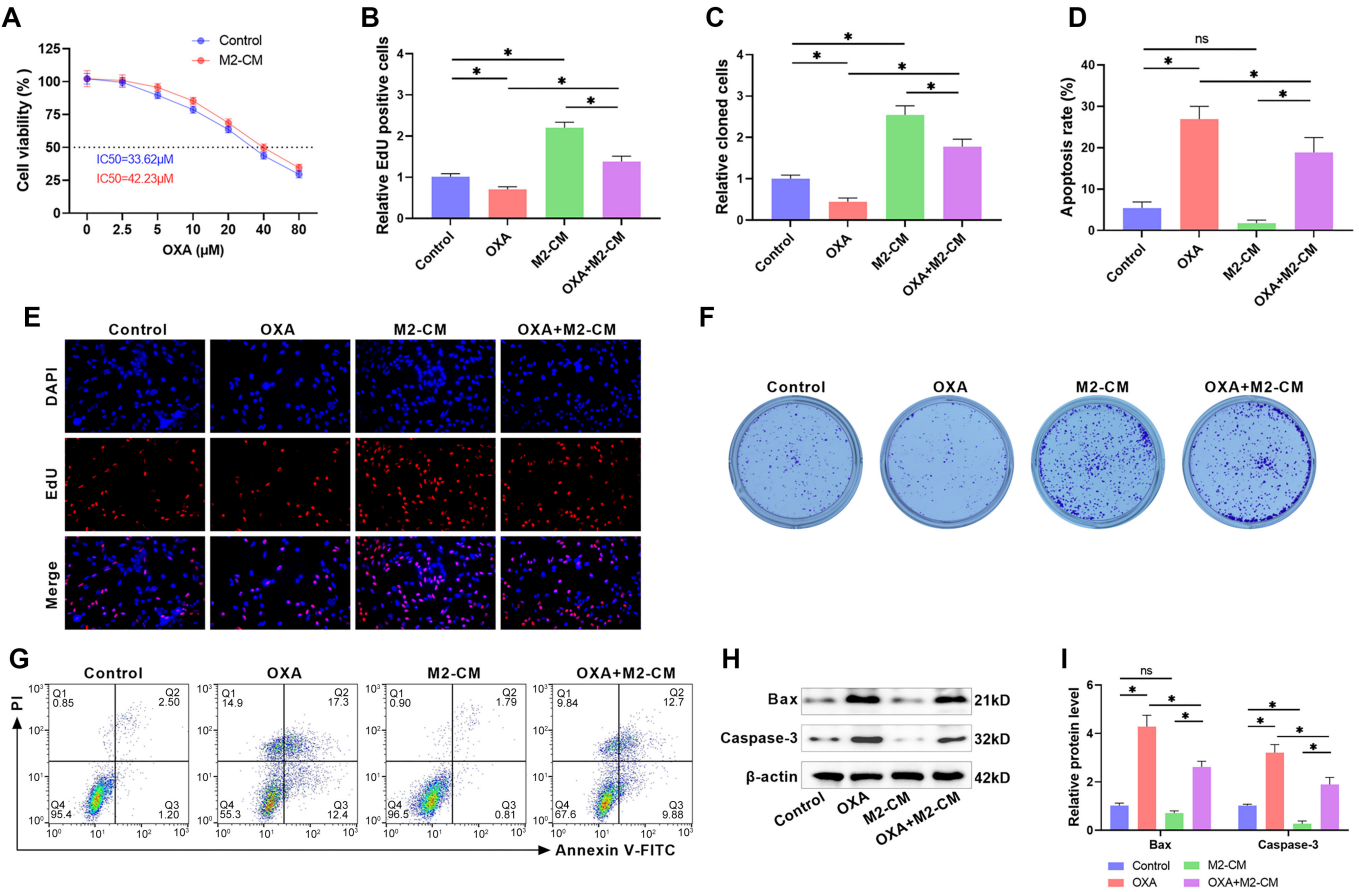
**M2 macrophages increase OXA resistance in HepG2/OXA cells.** (A) The IC50 of HepG2/OXA cells against OXA in the presence or absence of M2-CM was assessed using the CCK-8 assay; (B and E) EdU staining was used to detect the effect of OXA, M2-CM, or OXA+M2-CM co-treatment on the proliferation of HepG2/OXA cells; (C and F) Clone formation assay was used to examine the effects of OXA, M2-CM, or OXA+M2-CM co-treatment on clone formation in HepG2/OXA cells; (D and G) The apoptosis rate of HepG2/OXA cells after OXA, M2-CM, or OXA+M2-CM co-treatment was quantified using flow cytometry; (H and I) Western blot was used to quantify Bax and Caspase 3 levels in HepG2/OXA cells after OXA, M2-CM, or OXA+M2-CM co-treatment. M2-CM: M2 macrophage-conditioned medium; HepG2/OXA: OXA-resistant HepG2 cell line; OXA: Oxaliplatin.

To examine the potential effect of M2 macrophages on the sensitivity of HepG2/OXA cells to OXA, we supplemented the cell culture system with M2-CM and exposed the cells to 10 µM OXA for 48 h. Following the addition of M2-CM, the IC50 value of HepG2/OXA cells against OXA increased to 42.23 µM, as revealed by the CCK-8 assay results (Figure 3A). EdU staining showed that OXA treatment significantly reduced EdU positivity in HepG2/OXA cells, whereas the inhibitory effect of OXA on the proliferation of HepG2/OXA cells was attenuated by the addition of M2-CM to the culture system (Figure 3B and 3E). Additionally, OXA treatment notably diminished the number of clones in HepG2/OXA cells, but the suppressive effect of OXA on the clone-forming ability of HepG2/OXA cells was alleviated by the addition of M2-CM (Figure 3C and 3F). The apoptosis rate of HepG2/OXA cells was examined by flow cytometry and it revealed that OXA+M2-CM treatment attenuated the apoptosis rate in HepG2/OXA cells compared to OXA treatment alone (Figure 3D and 3G). Western blot analysis showed that the levels of Bax and Caspase-3 in HepG2/OXA cells were significantly elevated after OXA treatment, whereas the addition of M2-CM weakened the effect of OXA (Figure 3H and 3I). These findings indicate that M2 macrophages attenuate the impact of OXA and increase OXA resistance in HepG2/OXA cells.

### PPLN inhibits M2 macrophage polarization

**Figure 4.**
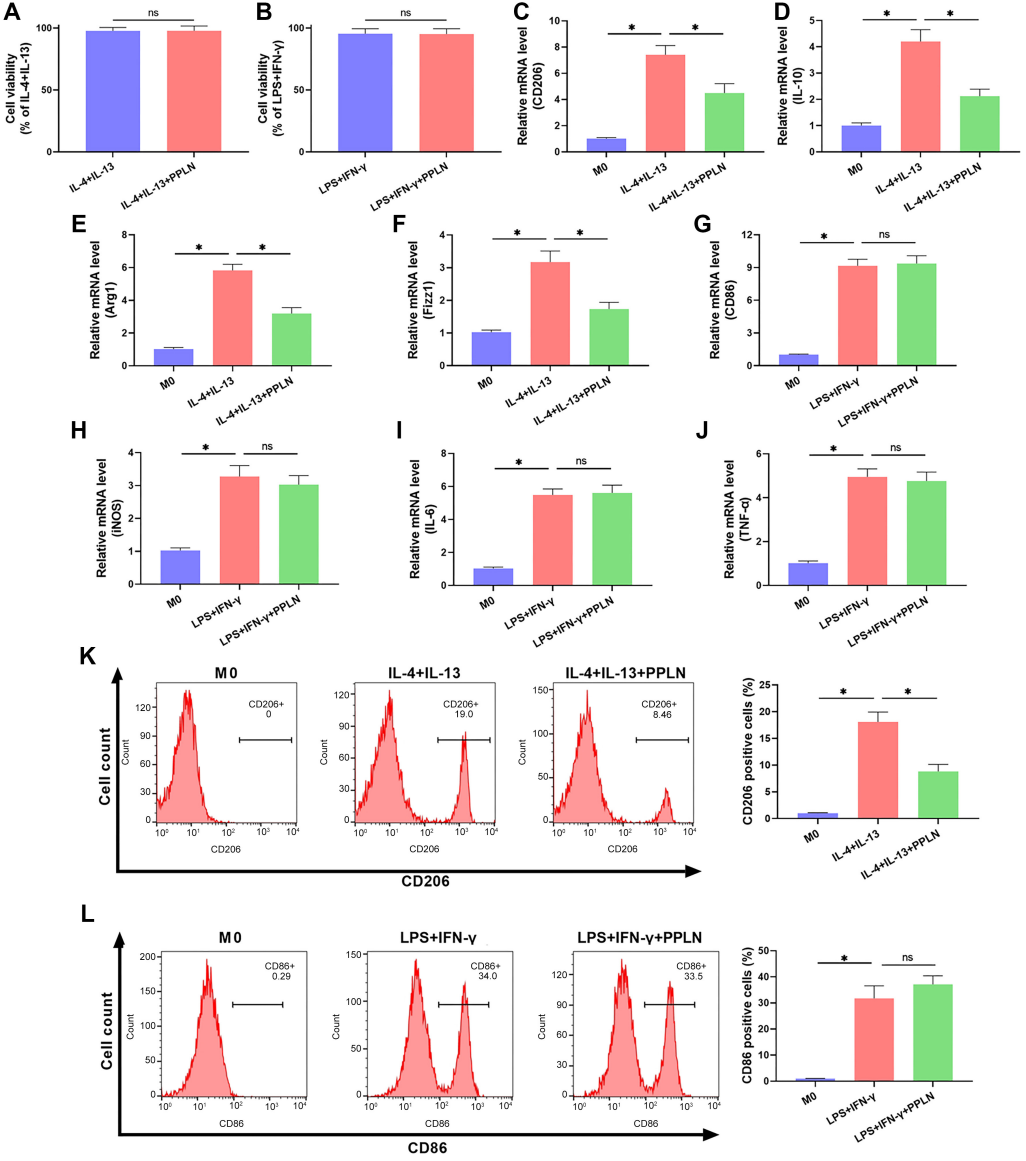
**PPLN inhibits M2 macrophage polarization.** (A and B) The effect of PPLN treatment on M1 and M2 macrophage viability was detected using the CCK-8 assay; (C–F) The levels of polarization markers CD206, IL-10, Arg1, and Fizz1 in M2 macrophages were quantified using RT-qPCR; (G–J) The levels of polarization markers CD86, iNOS, IL-6, and TNF-α in M1 macrophages were examined using RT-qPCR; (K) The expression of CD206, a polarization marker in M2 macrophages, was detected by flow cytometry; (L) Expression of CD86, a marker of M1 macrophage polarization, detected by flow cytometry. PPLN: Periplocin.

The CCK-8 assay results showed that PPLN treatment had no significant effect on the viability of M1 or M2 macrophages (Figure 4A and 4B). Next, we examined the levels of markers for M2 and M1 macrophage polarization following PPLN treatment to explore its influence on macrophage polarization. The results of RT-qPCR indicated a significant increase in the expression of M2 macrophage polarization markers CD206, IL-10, Arg1, and Fizz1 after treatment with IL-4+IL-13, whereas PPLN decreased the levels of these markers, suggesting that PPLN inhibits M2 macrophage polarization (Figure 4C–4F). In contrast, the levels of M1 macrophage polarization markers CD86, iNOS, IL-6, and TNF-α were significantly elevated after LPS+IFN-γ treatment, and PPLN did not cause noticeable changes in the expression of these markers, indicating that PPLN does not affect the polarization of M0 macrophages into M1 (Figure 4G–4J). Flow cytometry results similarly showed that PPLN treatment significantly reduced the number of CD206-positive cells, whereas there was no significant change in the number of CD86-positive cells, further confirming that PPLN inhibits M2 macrophage polarization but does not affect M1 macrophage polarization (Figure 4K and 4L).

### PPLN enhances OXA sensitivity of HepG2/OXA cells by hindering M2 macrophage polarization

**Figure 5.**
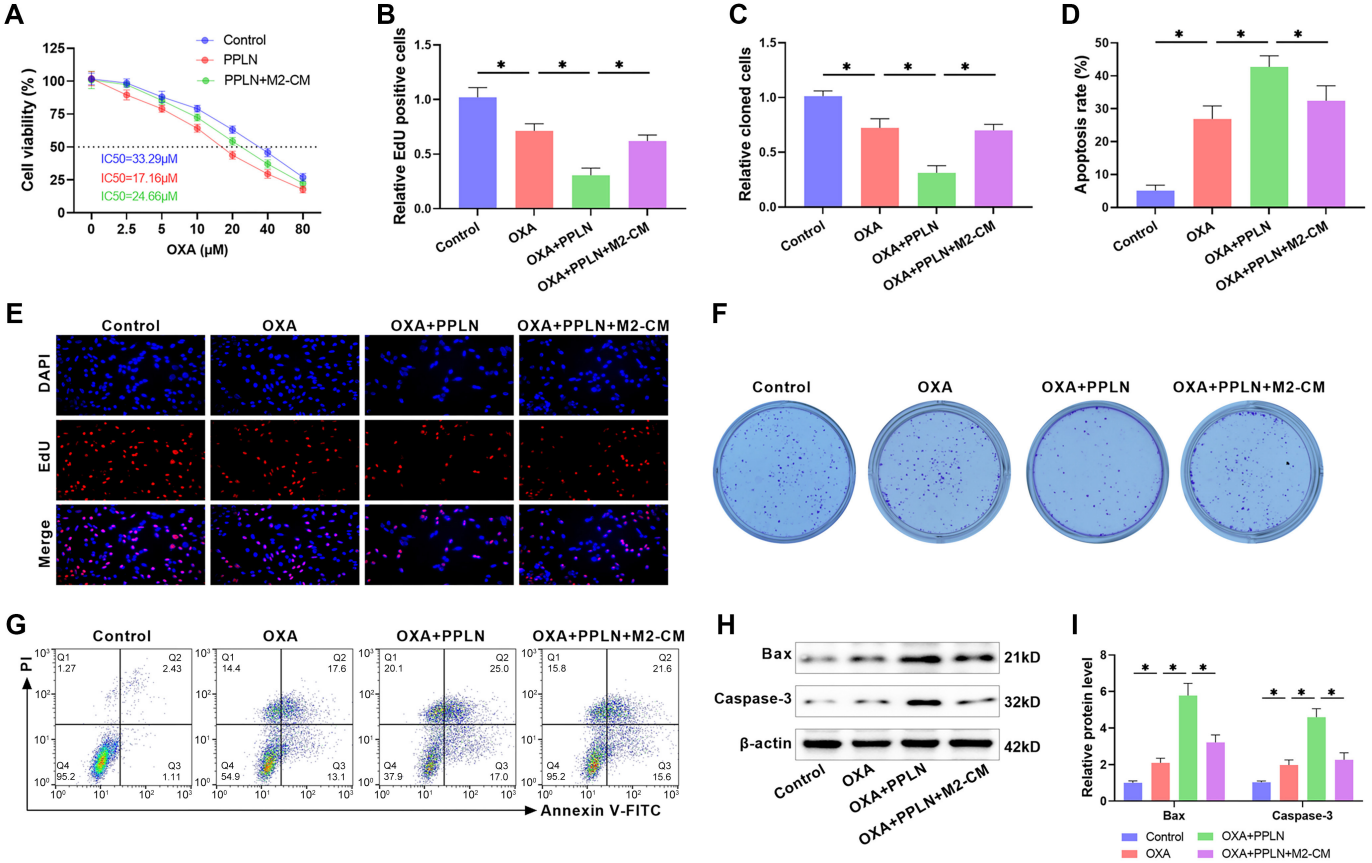
**PPLN enhances OXA sensitivity of HepG2/OXA cells by inhibiting M2 macrophage polarization.** (A) CCK-8 assay was used to assess the IC50 values of OXA in HepG2/OXA cells after PPLN or PPLN+M2-CM treatment; (B and E) The proliferation of HepG2/OXA cells treated with OXA, OXA+PPLN, or OXA+PPLN+M2-CM was detected using EdU staining; (C and F) Clone formation assay was used to detect clone formation in HepG2/OXA cells after OXA, OXA+PPLN, or OXA+PPLN+M2-CM treatment; (D and G) The apoptosis rate of HepG2/OXA cells treated with OXA, OXA+PPLN, or OXA+PPLN+M2-CM was examined using flow cytometry; (H and I) The levels of Bax and Caspase 3 in HepG2/OXA cells after OXA, OXA+PPLN, or OXA+PPLN+M2-CM treatment were detected using Western blot. PPLN: Periplocin; HCC: Hepatocellular carcinoma; HepG2/OXA: OXA-resistant HepG2 cell line; OXA: Oxaliplatin; M2-CM: M2 macrophage-conditioned medium.

To determine whether PPLN enhances OXA sensitivity in HepG2/OXA cells by hindering M2 macrophage polarization, we added PPLN and M2-CM to the HepG2/OXA cell culture system and assessed cell viability using the CCK-8 assay. After PPLN treatment, the IC50 value of HepG2/OXA cells against OXA decreased to 17.16 µM, whereas the IC50 value increased to 24.66 µM in the simultaneous presence of PPLN and M2-CM, suggesting that M2-CM attenuated the enhancing effect of PPLN on the chemosensitivity of HepG2/OXA cells (Figure 5A). EdU staining revealed a significant attenuation in EdU fluorescence intensity and a notable decline in the number of EdU-positive cells in OXA+PPLN-treated HepG2/OXA cells, whereas the addition of M2-CM attenuated the effect of OXA+PPLN (Figure 5B and 5E). Similarly, we found that OXA+PPLN treatment reduced the clone-forming ability of HepG2/OXA cells, which was reversed by the addition of M2-CM (Figure 5C and 5F). Flow cytometry results demonstrated that OXA+PPLN treatment led to a marked increase in the apoptosis rate of HepG2/OXA cells, whereas the addition of M2-CM attenuated the apoptosis-promoting effect of OXA+PPLN (Figure 5D and 5G). Moreover, the levels of Bax and Caspase-3 were significantly elevated in HepG2/OXA cells after OXA+PPLN treatment, whereas the addition of M2-CM reduced this effect (Figure 5H and 5I). These findings suggest that M2 macrophage polarization attenuates the effect of OXA+PPLN, indicating that PPLN improves the sensitivity of HepG2/OXA cells to OXA by hindering M2 macrophage polarization.

### PPLN enhances OXA sensitivity in HepG2/OXA cells *in vivo*

**Figure 6.**
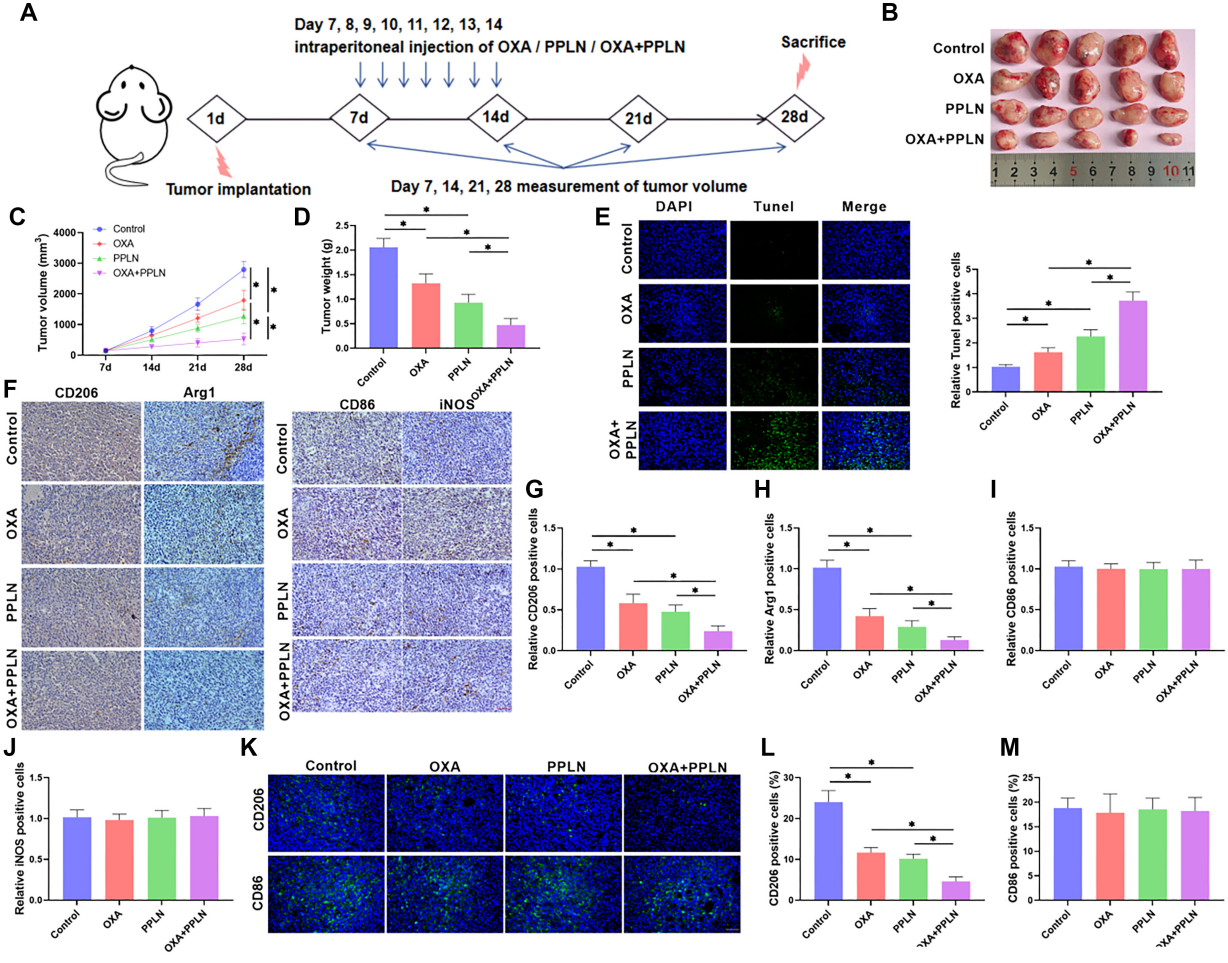
**PPLN enhances OXA sensitivity in HepG2/OXA cells *in vivo*.** (A) The process of constructing the nude mouse subcutaneous graft tumor model; (B) The effect on tumor growth after subcutaneous injection of OXA (10 mg/kg), PPLN (15 mg/kg), or OXA+PPLN compared to the control group (*n* ═ 5, day 28); (C) Measurement of subcutaneous grafted tumor volumes in nude mice at 7, 14, 21, and 28 days after different treatments; (D) Measurement of tumor weights in nude mice after different treatments (day 28); (E) Tunel staining was used to examine apoptosis in tumor tissues of nude mice; (F–J) The levels of M2 macrophage markers (CD206 and Arg1) and M1 macrophage markers (CD86 and iNOS) in tumor tissues were assessed using immunohistochemistry; (K–M) Immunofluorescence detected the expression of the M2 macrophage marker CD206 and the M1 macrophage marker CD86 in tumor tissues. PPLN: Periplocin; HepG2/OXA: OXA-resistant HepG2 cell line; OXA: Oxaliplatin; Arg1: Arginase-1; iNOS: Inducible nitric oxide synthase.

Finally, a nude mouse xenograft tumor model was constructed according to the process shown in Figure 6A to explore the impact of OXA and PPLN on tumor growth *in vivo*. The results showed that the injection of either OXA or PPLN significantly reduced tumor volume and weight in nude mice, *in vivo*. The combined OXA+PPLN injection resulted in a more pronounced inhibition of tumor growth compared to individual treatments (Figure 6B–6D). In addition, Tunel staining revealed a marked increase in the number of Tunel-positive cells in tumor tissues following the injection of OXA or PPLN, which was further elevated after the injection of OXA+PPLN (Figure 6E). We used immunohistochemistry to assess the expression of M2 and M1 macrophage polarization markers in HCC tissues. The results revealed that M2 macrophage polarization markers CD206 and Arg1 were notably down-regulated by the injection of OXA or PPLN and were further reduced by the injection of OXA+PPLN (Figure 6F–6H). In contrast, the injection of OXA, PPLN, or OXA+PPLN had no significant effect on the expression of M1 macrophage markers CD86 and iNOS (Figure 6I and 6J). Furthermore, immunofluorescence results showed that the injection of OXA or PPLN significantly reduced the number of CD206-positive cells, and OXA+PPLN treatment further reduced it. However, none of these treatments affected the number of CD86-positive cells (Figure 6K–6M). These results further indicated that PPLN hindered M2 macrophage polarization *in vivo*, consequently increasing the sensitivity of HepG2/OXA cells to OXA.

## Discussion

Continuous OXA chemotherapy often leads to drug resistance in HCC cells, severely limiting its efficacy in clinical applications [29, 30]. Exploring safe drugs for chemosensitization has positive implications for the treatment of HCC patients. Drug resistance can be categorized into acquired and natural resistance depending on its cause, with acquired resistance often resulting from the continuous exposure of cells to chemotherapeutic drugs on a continuous basis [31, 32]. Other studies have indicated that several biological processes, including drug efflux pumps, DNA damage repair, apoptosis, hypoxia, and epigenetic modifications, are associated with OXA resistance [33]. Therefore, establishing OXA-resistant cell lines *in vitro*, focusing on enhancing the OXA sensitivity of HCC cells, and exploring the underlying mechanisms will help address the challenge of OXA’s limited efficacy. Through a gradual increase in drug concentration, we developed an OXA-resistant HepG2/OXA cell line in this research. The proliferation-inhibiting and apoptosis-promoting effects of OXA on HepG2 cells were significantly stronger than on HepG2/OXA cells, confirming that the construction of the HepG2/OXA drug-resistant cell line was successful.

In recent years, the role of herbal extracts in inhibiting the malignant progression of tumors has attracted much attention because of their safety, low toxicity, and side effects, multi-pathway, and multi-target characteristics [34, 35]. As the main extract of Periplocae, PPLN has strong antitumor activity. Zhao et al. [36] showed that PPLN promoted apoptosis in gastric cancer cells through the ERK1/2-EGR1 pathway, and the combination of PPLN and TRAIL resulted in a greater reduction in cell viability compared to individual treatments, suggesting that PPLN increased the TRAIL sensitivity of gastric cancer cells. We found that PPLN dose-dependently decreased the activity of HepG2/OXA cells, lowered the IC50 value of HepG2/OXA cells for OXA, and enhanced the effect of OXA in hindering proliferation and inducing apoptosis both *in vivo* and *in vitro*, implying that it could improve the OXA sensitivity of HCC cells, which is similar to previous findings.

Macrophages alter their phenotype to restore homeostasis in the body and enhance their ability to cope with changes in their surroundings, a phenomenon referred to as macrophage polarization [37]. Diversity and plasticity are classical features of macrophages, which differentiate into two classical phenotypes: M1 and M2. M1 macrophages are classically activated macrophages with pro-inflammatory properties, whereas M2 macrophages are selectively activated macrophages with anti-inflammatory properties [38]. Exposure to pathogen-associated molecules, such as IFN-γ, TNF-α, or LPS causes macrophages to polarize to the M1 type, which secrete abundant quantities of pro-inflammatory factors, dominate the inflammatory microenvironment unfavorable to tissue recovery, and participate in anti-tumor immunity [39, 40]. In contrast, exposure to IL-4, IL-10, glucocorticoids, TGF-β, or immune complexes triggers M2 macrophage polarization [41]. M2 macrophages release a variety of chemokines and anti-inflammatory substances to avoid excessive damage to the body and promote wound healing [42]. M1 macrophages highly express CD80, iNOS, CD86, IL-6, and TNF-α, compared with elevated expression of Fizz1, Arg-1, CD206, IL-10, CCL17, and CCL22 in M2 macrophages [43–45]. Notably, our results showed that PPLN reduced the levels of M2 macrophage polarization markers both *in vivo* and *in vitro*, with no notable impact on the expression of M1 polarization markers, suggesting that PPLN inhibits M2 macrophage polarization.

Evidence is mounting to support the idea that M2 macrophage polarization is a significant factor in fueling the progression of malignant tumors [46, 47]. Chen et al. [48] reported that CHI3L1 protein released by M2 macrophages promoted metastasis of gastric and breast cancer cells. Yu et al. [49] indicated that M2 macrophage-derived exosomes circ 0008253 reduced the impact of OXA on gastric cancer cells and decreased the apoptosis rate, suggesting that M2 macrophage-derived exosomes enhance OXA resistance in gastric cancer cells. Similarly, Qu et al. [50] found that M2 macrophage polarization increased cisplatin resistance in gastric cancer cells. For this research, we induced M0 macrophage polarization into M2 macrophages to prepare M2-CM, mimicking tumor-associated M2 macrophage infiltration *in vitro*. We found that M2 macrophages attenuated the effect of OXA and increased the resistance of HepG2/OXA cells to OXA, consistent with previous findings. Furthermore, to determine whether PPLN enhances the sensitivity of HepG2/OXA cells to OXA by inhibiting M2 macrophage polarization, we added PPLN and/or M2-CM to the HepG2/OXA cell culture system. We found that the addition of M2-CM weakened the inhibitory effect of OXA+PPLN on cell proliferation and its promoting effect on cell apoptosis, thereby attenuating the chemosensitizing effect of PPLN. These results suggest that PPLN improves the OXA sensitivity of HepG2/OXA cells by hindering M2 macrophage polarization.

## Conclusion

PPLN enhanced the effect of OXA, decreased the viability and proliferation of HepG2/OXA cells, and promoted their apoptosis. In addition, M2 macrophage polarization increased the OXA resistance of HepG2/OXA cells, whereas PPLN inhibited M2 macrophage polarization. These findings suggest that PPLN enhances the OXA sensitivity of HepG2/OXA cells by hindering M2 macrophage polarization. This study elucidated the mechanism of PPLN in enhancing the chemosensitivity of HCC cells through *in vitro* and *in vivo* experiments, providing new insights for the clinical application of PPLN, the treatment of chemotherapy-resistant patients, and the improvement of their prognosis. However, there are still some shortcomings, and further exploration of the signaling pathways through which PPLN enhances the chemosensitivity of HCC cells is needed.

## Supplemental data

**Highlights:**

1. PPLN inhibited the proliferation of HepG2/OXA cells and promoted their apoptosis.
2. PPLN increased the effect of OXA and enhanced the OXA sensitivity of HepG2/OXA cells.
3. M2 macrophages increased OXA resistance in HepG2/OXA cells.
4. PPLN inhibited M0 macrophage polarization to M2 macrophages but had no influence on M1 polarization.
5. PPLN enhanced OXA sensitivity of HepG2/OXA cells *in vivo* and *in vitro* by hindering M2 macrophage polarization.

**Graphical abstract.**
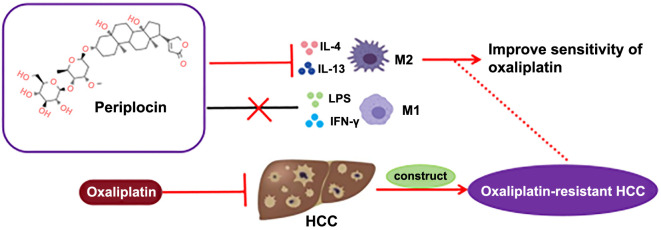
PPLN inhibits M2 macrophage polarization, suppresses proliferation, and promotes apoptosis of OXA-resistant HCC cells both *in vivo* and *in vitro*, thereby enhancing the sensitivity of drug-resistant cells to OXA.

## Data Availability

The data supporting the findings of this study can be obtained from the corresponding author, upon request.
